# Virus Infection-Induced Bronchial Asthma Exacerbation

**DOI:** 10.1155/2012/834826

**Published:** 2012-08-23

**Authors:** Mutsuo Yamaya

**Affiliations:** Department of Advanced Preventive, Medicine for Infectious Disease, Tohoku University Graduate School of Medicine, Sendai 980-8575, Japan

## Abstract

Infection with respiratory viruses, including rhinoviruses, influenza virus, and respiratory syncytial virus, exacerbates asthma, which is associated with processes such as airway inflammation, airway hyperresponsiveness, and mucus hypersecretion. In patients with viral infections and with infection-induced asthma exacerbation, inflammatory mediators and substances, including interleukins (ILs), leukotrienes and histamine, have been identified in the airway secretions, serum, plasma, and urine. Viral infections induce an accumulation of inflammatory cells in the airway mucosa and submucosa, including neutrophils, lymphocytes and eosinophils. Viral infections also enhance the production of inflammatory mediators and substances in airway epithelial cells, mast cells, and other inflammatory cells, such as IL-1, IL-6, IL-8, GM-CSF, RANTES, histamine, and intercellular adhesion molecule-1. Viral infections affect the barrier function of the airway epithelial cells and vascular endothelial cells. Recent reports have demonstrated augmented viral production mediated by an impaired interferon response in the airway epithelial cells of asthma patients. Several drugs used for the treatment of bronchial asthma reduce viral and pro-inflammatory cytokine release from airway epithelial cells infected with viruses. Here, I review the literature on the pathogenesis of the viral infection-induced exacerbation of asthma and on the modulation of viral infection-induced airway inflammation.

## 1. Introduction

Infection with respiratory viruses, including rhinovirus (RV), influenza, virus and respiratory syncytial (RS) virus, is the major cause of the common cold, and these infections exacerbate bronchial asthma [1–6]. Several mechanisms for the viral infection-induced exacerbation of asthma, including airway inflammation [7–10], mucus hypersecretion, and bronchial hyperresponsiveness [9, 11], have been reported. Viruses infect cells in the airways, such as epithelial cells and mast cells, and the infection induces the production of various pro-inflammatory cytokines [12–15] and mediators [16–19] (Table 1). RV infection also enhances smooth-muscle contractility [20, 21]. An impaired immune response may be correlated with a higher susceptibility to RV infection and viral replication in asthma patients and an increased severity of RV-induced exacerbation of bronchial asthma [22–24].

Several drugs, including systemic and inhaled corticosteroids and long-acting *β*_2_ agonists, have been developed, which are used for the treatment and prevention of asthma exacerbations and for the management of stable bronchial asthma [62]. Recent reports have demonstrated that corticosteroids reduce the release of inflammatory mediators from airway epithelial cells infected with RV [33–35] and that the combination of corticosteroids and *β*_2_ agonists have additive or synergistic effects. In contrast, whether these drugs inhibit viral infection is still uncertain. Our group has demonstrated that several drugs, including dexamethasone (a corticosteroid), procaterol (a *β*_2_ agonist), tiotropium (an anti-cholinergic agent), L-carbocisteine (a mucolytic agent), and macrolides, reduce the release of viruses and pro-inflammatory cytokines from human tracheal epithelial cells [36–40]. Here, I review the pathogenesis and management of the viral infection-induced exacerbation of bronchial asthma. Furthermore, I review the findings on the inhibitory effects of drugs that are used for the treatment of bronchial asthma and other inflammatory pulmonary diseases on viral infection and infection-induced inflammation.

## 2. The Cinical Importance of Viral Infection in Asthma Exacerbation

### 2.1. Association between Viral Infection and Asthma Exacerbation

 Infection by respiratory viruses, including RV, influenza virus, and RS virus, often provokes wheezing in patients with asthma [1–6]. Upper respiratory tract viral infections are associated with hospital admission for asthma [63], and the incidence of RV infection is suggested to be higher in patients with asthma compared with control subjects [3, 64]. Studies using reverse transcription PCR assays have demonstrated the importance of RVs, reporting that RVs are responsible for 80–85% and 45% of the asthma flairs in 9–11-year-old children and in adults, respectively [4, 5]. Recent studies have also demonstrated the importance of RVs and the RS virus in asthma exacerbation in children [65]. Infection with the 2009 pandemic influenza A (A/H_1_N_1_ 2009) virus also induced severe asthma exacerbation [66], especially in children.

### 2.2. Rhinovirus Replication in Patients with Bronchial Asthma

 To investigate the underlying mechanisms of the increased susceptibility to RV infection in patients with bronchial asthma [3, 64], Wark et al. [22] examined viral replication and the innate responses to RV infection in primary bronchial epithelial cells from asthmatic patients. They found that viral RNA expression and the release of viruses into the supernatant were increased in asthmatic cells compared with the healthy controls. They suggested that the impairment of virus-induced interferon (IFN)-*β* expression may be associated with enhanced viral replication in asthmatic cultures (Table 1(b)). Contoli et al. [23] also demonstrated that the deficient induction of IFN-lambda (IFN-*λ*) by RV was highly correlated with the severity of RV-induced asthma exacerbation, including the cold score and decreases in the forced expiratory volume in one second (FEV1), and with the virus load in asthmatic primary bronchial epithelial cells and alveolar macrophages in experimentally infected human volunteers. Iikura et al. [24] reported lower IFN-*α* production in peripheral blood mononuclear cells from asthmatic patients after RV infection. Thus, the reduction in these impaired innate responses may be associated with a higher susceptibility to RV infection. Enhanced viral replication may augment airway inflammation by recruiting neutrophils and potentially other inflammatory cells, causing increased mediator release and the exacerbation of bronchial asthma.

## 3. The Effects of Viral Infection on Human Subjects

### 3.1. Inflammatory Cells in the Airways during Rhinovirus Infection

RV infection causes an infiltration of neutrophils, lymphocytes and eosinophils in the nasal and bronchial mucosa [7–10]. Neutrophils accumulate in the airway during the acute stage of a cold [7, 8]. The levels of IL-8 (CXCL8), a chemokine, and myeloperoxidase in nasal aspirates increase in children during RV infection-induced asthma exacerbation [25] (Table 1(a)). Furthermore, the myeloperoxidase levels in nasal aspirates correlate with the severity of the upper respiratory symptoms [25]. Thus, IL-8 and myeloperoxidase secreted in the airway after RV infection may be associated with the onset of asthma exacerbation.

RV stimulates lymphocytes to induce IFN-*γ* production and T-cell proliferation through the activation of eosinophils [50] and monocytes [51]. Experimental RV infection studies revealed the accumulation of lymphocytes in the airway mucosa [10]. 

The plasma histamine content increases after RV infection [18]. RV infection increases the bronchial responsiveness to histamine in patients with bronchial asthma [11], and the provocative concentration of histamine decreases following an RV infection, which causes a 20% decrease in FEV_1_ (PC20). Increases in histamine hyperresponsiveness were associated with an increase in the number of submucosal lymphocytes [9]. Furthermore, RV infection increases bronchial responsiveness to histamine and the ragweed antigen in association with increases in histamine release from the peripheral blood leukocytes in patients with allergic rhinitis [52]. Increased histamine production and bronchial responsiveness to histamine may be responsible for asthma attacks in RV infection.

### 3.2. Inflammatory Markers in the Exhaled Air during Viral Infection

To monitor lung inflammation in patients with bronchial asthma and patients infected with viruses, noninvasive methods have been developed that involve the measurement of exhaled markers, including exhaled nitric oxide (NO) [67–71], carbon monoxide (CO) [72], volatile gases (e.g., ethane and pentane) [73, 74], and endogenous substances (e.g., inflammatory mediators, cytokines, and oxidants).

NO is generated from the guanidino nitrogen of L-arginine during its oxidation to L-citrulline by the enzyme NO synthase (NOS), which has constitutive (cNOS) and inducible (iNOS) isoforms that have been described [75]. Constitutive NOS, which is basally expressed in endothelial and neuronal cells, releases only small amounts of NO [76]. In contrast, the expression of iNOS in epithelial and several inflammatory cells can be induced by pro-inflammatory cytokines such as tumor necrosis factor (TNF)-*α* and IL-1*β*, the production of which is increased by RV and RS viral infection [14, 15, 61, 77], and the induction of iNOS results in relatively high levels of NO.

In patients with bronchial asthma, experimental RV inoculation increases the NO concentrations [11], and the histamine PC20 decreases following an RV infection (Table 1(a)). These findings suggest that RV infection increases the exhaled NO levels in asthmatics and that this increase is associated with a worsening of airway hyperresponsiveness to histamine. In contrast, Leung et al. [78] reported low NO concentrations, most likely caused by breathlessness from severe bronchoconstriction and by treatment with systemic corticosteroids. The measurement of NO is now widely and clinically used to monitor lung inflammation while determining the appropriate treatment for patients with asthma. 

CO is endogenously produced predominantly via the activity of heme oxygenase, which degrades heme to CO, iron, and bilirubin in the cells [79]. Exhaled CO concentrations are associated with eosinophilic airway inflammation [72], and CO concentrations in the exhaled air also indicate airway inflammation in subjects who have never smoked or who have stopped smoking for 3 months or longer [80]. Several reports have demonstrated the use of an exhaled CO analysis to monitor airway inflammation in patients with asthma and in patients infected with viruses [29, 30, 72, 81–83].

The CO concentrations in exhaled air are influenced by the CO concentrations in the inhaled air, and they cannot be used as an inflammatory marker in smokers. However, in subjects with upper respiratory tract infections (URTIs), such as seasonal type A influenza virus, the exhaled CO concentrations were found to be increased at the time of their URTI symptoms [29] (Table 1(a)). Furthermore, we measured the exhaled CO concentrations in 20 patients who were experiencing acute asthma exacerbation. Fourteen of the 20 patients were infected with the seasonal type A influenza virus [30]. Asthma exacerbation caused a reduction in the peak expiratory flow rate (PEFR) and an increase in exhaled CO in all patients. The CO concentrations were shown to be inversely correlated with PEFR in the treatment of asthma patients infected with URTIs, and treatment with oral glucocorticoids reversed the changes in both parameters [30]. These findings suggest that the exhaled CO concentration can be a marker of the development of exacerbation and of the efficacy of the treatment for asthma exacerbation by infection. 

## 4. Inflammatory Mediators during Viral Infection-Induced Asthma Exacerbation

**Figure 1:**
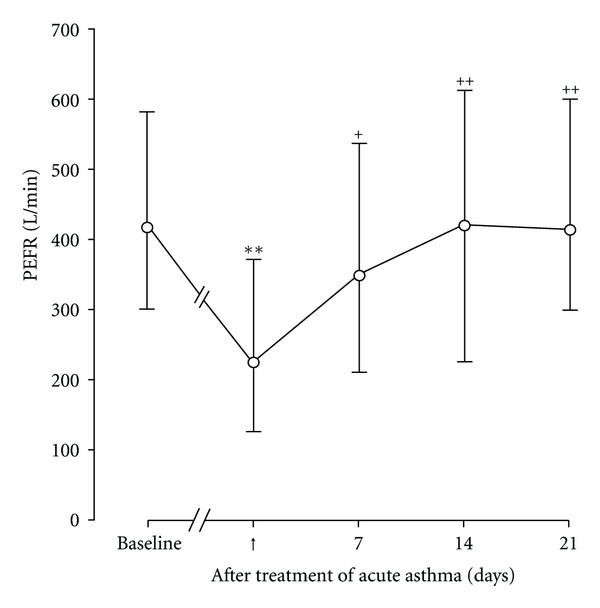
Time course changes in peak expiratory flow rate (PEFR) in asthmatic patients (*n* = 30) before acute asthma exacerbations (baseline) and after treatment with oral glucocorticoids. ↑: the start of treatment of acute asthma exacerbations with oral glucocorticoids. Means and ranges are indicated by open circles with bars. Significant differences from baseline are indicated by **P < 0.01. Significant differences from acute asthma exacerbations are indicated by ^+^ *P* < 0.05 and ^++^ *P* < 0.01. (Cited from [19]).

**Figure 2:**
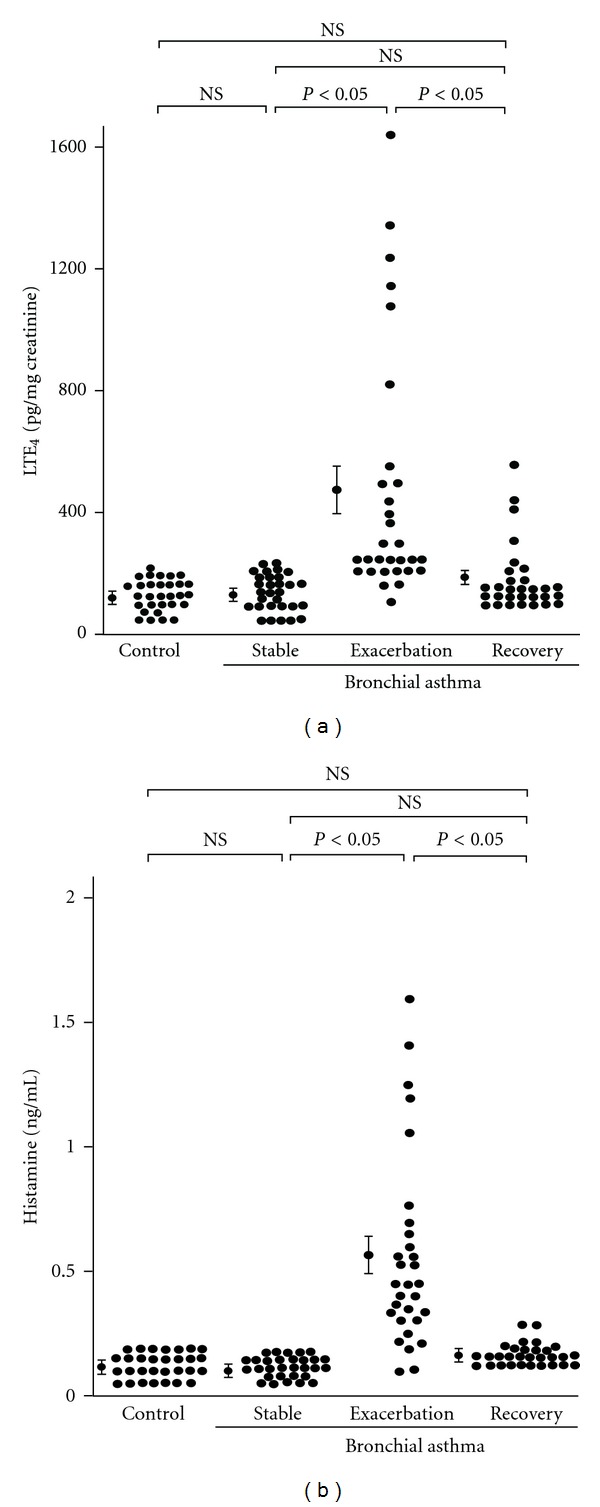
Concentrations of urinary LTE_4_ (a) and plasma histamine (b) in control subjects (Control, *n* = 30) and patients with bronchial asthma (*n* = 30) at a stable condition (Stable), during exacerbations (Exacerbation) and after 21 days of treatment with oral glucocorticoids when patients showed evidence of clinical improvement (Recovery). Mean values ± S.E.M. are indicated by closed circles with error bars. NS, not significant. (Cited from [19]).

**Figure 3:**
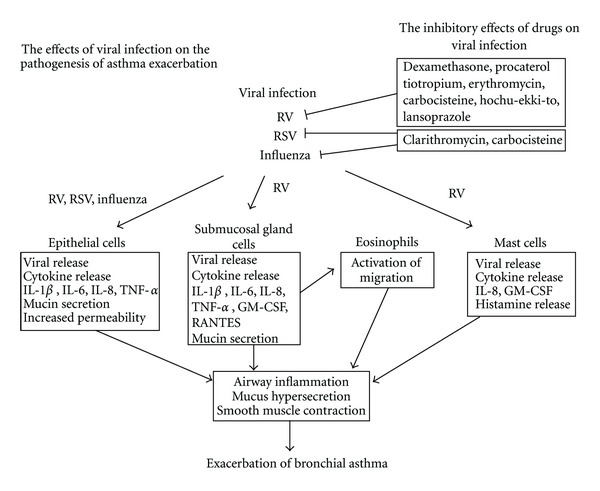
Summary of our findings on the effects of viral infection on the pathogenesis of viral infection-induced asthma exacerbation and on the inhibitory effects of drugs on viral infection. RV: rhinovirus, RSV: respiratory syncytial virus, IL: interleukin, TNF: tumor necrosis factor, GM-CSF: granulocyte-macrophage colony stimulating factor. (Cited from [14, 15, 19, 29, 30, 36–40, 43–46, 49, 53, 55, 56, 61, 72, 77, 80, 84]).

To examine the mechanisms of viral infection-induced asthma exacerbation, we studied the relationship between airway narrowing and the inflammatory and bronchospastic factors in the peripheral venous blood and urine of 30 patients with asthma exacerbation caused by URTIs [19]. The acute asthma exacerbation caused decreases in the PEFR in all 30 patients with asthma (Figure 1). The asthmatic exacerbation resulted in increased serum levels of IL-6, soluble intercellular adhesion molecule-1 (sICAM)-1 and ECP, and increased concentrations of urinary leukotriene E_4_ (LTE_4_) and plasma histamine compared with patients with stable asthma and with the 30 control subjects (Figures 2 and 3, Table 1(a)). At the onset of URTI symptoms, RV was identified in 13 patients, and seasonal type A influenza virus was detected in 7 patients. In addition, the parainfluenza virus, adenovirus and enterovirus were each identified in 1 patient. In patients with RV infection during the exacerbations, the serum levels of IL-6, sICAM-1 and ECP, and the concentrations of urinary LTE_4_ and plasma histamine were lower than those in patients infected with viruses other than RV during the exacerbations, including the influenza virus and adenovirus [19]. During asthma exacerbation, the PEFR values negatively correlated with the levels of these factors. Treatment with oral glucocorticoids reversed the decreases in the PEFR and the increases in these factors. These findings suggest that respiratory viral infections may cause acute asthma exacerbation via the production of mediators that induce inflammation and bronchospasm. RV infection has also been shown to increase the IL-6 concentrations in the nasal secretions [13], and increased serum levels of IL-6 were reported in children with acute respiratory infections [31]. An experimental RV infection increased the plasma histamine concentrations after an antigen challenge in asthmatic patients [18]. Similarly, IL-11 was detected in the nasal aspirates from children with viral URTIs [32], and the IL-11 levels were highest in patients with clinically detectable wheezing. Thus, pro-inflammatory cytokines, inflammatory mediators and substances in the airway mucosa and submucosa, including IL-6, IL-11, ICAM-1, ECP, LTC_4_, LTD_4_, and histamine, may induce airway inflammation and smooth muscle contraction in asthma patients with viral infections.

Similarly, eosinophil accumulation is observed in the airway mucosa [10] after experimental RV infection. Eosinophil granular proteins, including the eosinophil cationic protein (ECP), have also been detected in the nasal secretions of children with wheezing symptoms caused by RV infection [26] and in the sputum of asthmatic patients experimentally infected with type 16 RV (RV16) [27]. The stimulation of eosinophil and T-cell proliferation may cause the airway inflammation and subsequent smooth muscle contraction caused by RV infection. 

## 5. The Effects of Viral Infection on Airway Epithelial Cells

To understand the mechanisms of airway inflammation and asthma exacerbation after viral infection, various studies have been performed to examine the production of pro-inflammatory substances, adhesion molecules and chemical mediators in lung cells. RV infection increases the production of various pro-inflammatory substances, including IL-1*α*, IL-1*β*, IL-6, IL-8 (CXCL8), IL-11, TNF-*α*, regulated on activation normal T cell expressed and secreted (RANTES; CCL5 = CC Chemokine Ligand 5), and granulocyte-macrophage colony stimulating factor (GM-CSF), in primary cultures of epithelial cells or cell lines (Table 1(b)).

Subauste et al. [12] demonstrated that RV infection induced the release of IL-6, IL-8 and GM-CSF from a human bronchial epithelial cell line (BEAS-2B) and that the preexposure of BEAS-2B cells to TNF-*α* increased the susceptibility of the cells to RV infection. The authors suggested that inflammatory cytokines produced by RV infection may increase the susceptibility to RV infection. IL-6 induces antibody production in B cells and also T-cell activation and differentiation [85]. IL-8 is a major chemoattractant for neutrophils, and it stimulates these cells to release enzymes and produce reactive oxygen species [86]. Similarly, GM-CSF can prime both neutrophils and eosinophils for enhanced activation to chemical stimuli [87].

Major-type RVs, including RV14 and RV16, and minor type RVs, including RV1 and RV2, can infect cultures of human tracheal and bronchial epithelial cells, tracheal submucosal gland cells, and alveolar epithelial cells by binding to the ICAM-1 and low-density lipoprotein (LDL) receptors, respectively, and they produce proinflammatory cytokines, including IL-1*α*, IL-1*β*, IL-6, IL-8, IL-11, TNF-*α*, RANTES, and GM-CSF and the ICAM-1 and LDL receptors [12, 14, 15, 22, 23, 32, 41–43] (Figure 3, Table 1(b)). RV infection also induces mucin secretion in epithelial cells [49]. The activation of the transcription factor nuclear factor-kappa (NF-*κ*) B is associated with the production of pro-inflammatory cytokines and ICAM-1 [13, 88, 89], and the endogenous production of IL-1*β* is also associated with ICAM-1 expression after RV infection [14].

The upregulation of ICAM-1, the receptor for the major group of rhinoviruses (RVs) [90, 91], was shown to increase cell susceptibility to the major group of RVs [12], which could lead the adjacent cells to become infected when the viruses are released from the originally infected cells. Chronic antigen challenge has been shown to increase ICAM-1 expression in the airway epithelium, which may be related to airway inflammation in asthma [92]. Inflammatory conditions such as asthma, in which ICAM-1 expression is increased on the respiratory epithelial surfaces, may cause a predisposition to RV infection because ICAM-1 is a receptor for the major group of RVs [90, 91].

IL-13 has been shown to increase in bronchial tissues from patients with asthma [93]. The cellular source of IL-13 was identified in the mononuclear cell fraction of the allergen-challenged bronchoalveolar lavage (BAL) [93]. Furthermore, Lachowicz-Scroggins et al. [94] demonstrated that IL-13 induced mucous metaplasia and increased the susceptibility of human airway epithelial cells to RV infection through a marked decrease in the ciliation and flatness of the mucosal side surface [94].

 Similar to RV infection, influenza viral infection induces the NF-*κ*B-mediated release of cytokines, including IL-1*β*, IL-6, and IL-8, from human tracheal epithelial cells [55, 56] (Figure 3, Table 1(b)). Increases in cytokines and monokines, including IL-6, IL-8, and RANTES, are also observed in the sera of patients infected with the influenza virus [28] (Table 1(a)). Similarly, RS viral infection induces the release of IL-1*β*, IL-6, and IL-8 from human airway and alveolar epithelial cells [32, 59–61] (Table 1(b)).

 Viral infection affects the barrier function of airway epithelial cells and vascular endothelial cells. We demonstrated that hydrogen peroxide increases the transepithelial influx of mannitol in cultured human tracheal epithelial layers and that RV infection further increases mannitol influx in cells treated with IL-1*β* [84] (Figure 3). These findings suggest that RV infection may affect the integrity of airway epithelial cells, although RV infection does not induce airway epithelial cell damage [95], unlike influenza viral infection [96].

Although the exact roles and potency of these effects are still uncertain, these pro-inflammatory cytokines, monokines, and inflammatory substances that are produced in airway epithelial cells may contribute to the development of airway inflammation, damaging the barrier function and leading to a subsequent asthma attack.

## 6. The Effects of Viral Infection on Cells Other Than Airway Epithelial Cells

 The mechanisms for viral infection-induced mucosal edema have been unclear; however, Wang et al. demonstrated that influenza virus infection increased the vascular endothelial permeability in mouse lungs through increased levels of IL-1*β*, IL-6, TNF-*α*, and trypsin [57] (Table 1(b)). 

**Figure 4:**
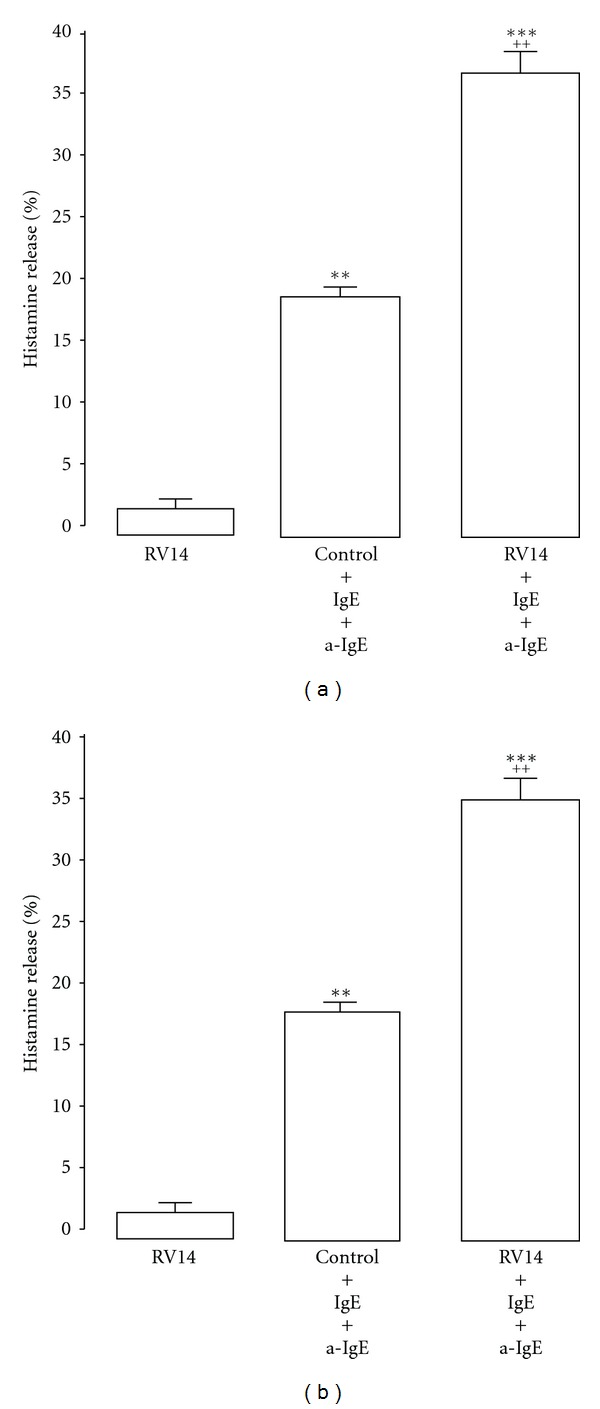
Histamine release into supernatants of the cell lines from human mast cells (HMC-1) (left side) and basophils (KU812) (right side) in the presence of immunoglobulin E (IgE) plus anti-IgE (IgE + a-IgE) after type 14 rhinovirus (RV14) or sham (control) infection. Results are reported as means ± S.E.M. from 7 samples. Significant differences from RV14 infection alone are indicated by **P < 0.01 and ***P < 0.001. Significant differences from stimulation with IgE + a-IgE alone are indicated by ^++^ *P *< 0.01 (Cited from [53]).

Cells other than lung epithelial cells have also been reported to produce pro-inflammatory substances and chemical mediators, such as histamine. Infection with respiratory viruses, including RVs, type A influenza virus and RS virus, activates histamine release from peripheral blood basophils [17]. Type A influenza virus infection increases histamine release from basophils that have been stimulated with anti-immunoglobulin E (IgE) and calcium ionophore [16, 58]. Mast cells are major sources of histamine release in the airways, and they are associated with the pathogenesis of bronchial asthma [97]. Hosoda et al. reported that RV infection primes the production of IL-4, IL-6, IL-8, GM-CSF, and histamine in response to stimuli such as IgE in both a human mast cell line and a human basophilic leukocyte cell line [53] (Figures 3 and 4, Table 1(b)).

Airway macrophages secrete TNF-*α* after RV infection [54]. TNF-*α* increases the expression of ICAM-1 and other adhesion molecules on a number of different cell types [98], and it is associated with wheezing illnesses in infancy [99] and the development of the late-phase allergic reactions and asthma [100].

Eosinophil accumulation is observed in the airway mucosa [10] after experimental RV infection. ECP has also been detected in the nasal secretions of children with wheezing symptoms caused by RV infection [26], in the sputa of asthmatic patients experimentally infected with RV16 [27], and in the sera of patients with URTIs who experience asthma exacerbation [19] (Table 1(a)). Increases in the ECP levels and the percentage of eosinophils in the sputum were correlated with airway hyperresponsiveness [27]. RV16 did not induce superoxide production by peripheral blood eosinophils, as shown by Handzel et al. [50], but Furukawa et al. [44] reported that human tracheal submucosal gland cells may augment eosinophil transmigration across the airway epithelium through the secretion of RANTES and GM-CSF after RV infection and may contribute to the exacerbations of asthma (Figure 3, Table 1(b)). Thus, inflammatory mediators, such as RANTES and GM-CSF [15, 41, 42, 44, 53], may be released from cells, such as airway submucosal cells and mast cells, and may activate eosinophils after RV infection. 

Furthermore, the direct effects of RV infection on airway smooth muscle contraction were demonstrated by Hakonarson et al. [20]. RV infection increased rabbit and human airway smooth muscle constrictor responsiveness to acetylcholine and attenuated the dose-dependent relaxation of the smooth muscle to *β*-adrenoceptor stimulation with isoproterenol [20]. IL-1*β*, which might be released from airway epithelial cells [14, 15] and smooth muscle [21], may enhance airway smooth muscle contraction in response to acetylcholine during RV infection through autocrine mechanisms.

Influenza virus infection-induced endothelial cell damage may be involved with the mucosal edema associated with airway inflammation. Furthermore, the production of pro-inflammatory cytokines and mediators and the production of inflammatory substances such as ECP in cells other than epithelial cells may also be related to airway hyperresponsiveness in asthma patients infected with respiratory viruses. RV infection may stimulate smooth muscle contraction in combination with mediators, such as leukotrienes and histamine, which are released from cells other than epithelial cells in the airways.

## 7. The Role of Toll-Like Receptor Activation in the Viral Infection-Induced Asthma Exacerbation

 Leukocytes, including dendritic cells, macrophages, lung epithelial cells and airway smooth muscle express Toll-like receptors (TLRs), including TLR2, TLR3 and TLR4 [101–103]. Infection with respiratory viruses, such as RV and RS virus activates these cells via the activation of TLRs, and induces mucus production and the secretion of various pro-inflammatory cytokines and monokines, including IL-6, IL-8, and RANTES [101–104]. Furthermore, the RV-induced IL-6 release was significantly greater in human airway smooth muscle cells derived from asthmatic subjects compared with nonasthmatic subjects [105]. RV infection augments airway smooth muscle contraction mediated by the release of inflammatory cytokines [21]. The viral infection-induced activation of TLRs may also contribute to the mechanisms for the exacerbation of bronchial asthma.

## 8. Modulation of Viral Infection and the Infection-Induced Release of Pro-Inflammatory Cytokines

### 8.1. Inhibition of Rhinovirus Infection and Infection-Induced Mediator Release

An effective vaccination for RV has not been developed because there are more than 100 RV serotypes. Furthermore, in contrast to the development of antiinfluenza virus drugs, antiviral drugs have not been developed for RVs and RS viruses. A variety of anti-viral agents have been studied for their ability to inhibit RV infection or the common cold, including vitamin C [106, 107], zinc gluconate lozenges [108, 109], WIN compounds [110, 111], soluble ICAM-1 [112, 113], RV 3C protease inhibitors [114], compound R77975 [115] and IFN-*α* [116]. However, the clinical benefits of these agents have not been established.

On the basis of the recent findings presented below, the modulation of NF-*κ*B is a promising target for the development of anti-inflammatory therapies for the asthma exacerbation induced by respiratory viral infections [117].

Inhaled corticosteroids and long-acting *β*_2_ agonists reduce the frequency of asthma exacerbation [62]. With regard to the effects of inhaled corticosteroids and long-acting *β*_2_ agonists on RV infection and the infection-induced production of pro-inflammatory cytokines, Skevaki et al. [35] demonstrated that the corticosteroid budesonide inhibits the production of inflammatory mediators, including IL-6, IL-8, RANTES and CXCL10 (= interferon gamma inducible protein-10, IP-10), in BEAS-2B cells, and primary human bronchial epithelial cells whereas the long-acting *β*_2_ agonist formoterol has no effect on the release of IL-6 (Table 2). The combination of budesonide and formoterol had additive or synergistic effects in the suppression of RV-induced IL-8, RANTES and IP-10. However, the authors did not show the data on the viral release from the cells. 

Edwards et al. reported that a combined treatment with a long-acting *β*_2_ agonist, salmeterol, and an inhaled corticosteroid, fluticasone, inhibited the RV-induced RANTES and IL-8 production in BEAS-2B cells compared with fluticasone alone [33]. They also demonstrated the inhibitory effects of fluticasone on the RV infection-induced IL-6 production in BEAS-2B cells and in primary bronchial epithelial cells [34].

**Figure 5:**
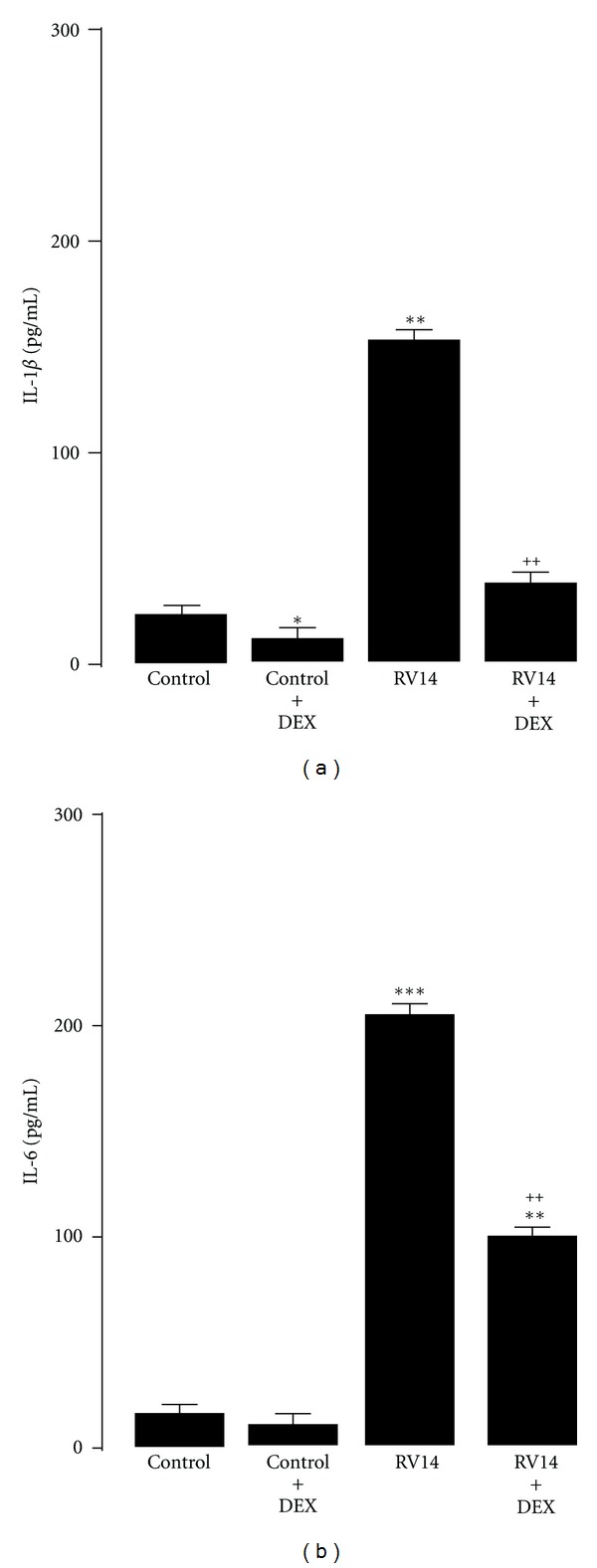
Effects of dexamethasone (DEX, 1 *μ*M) on release of IL-1*β* (a) and IL-6 (b) in supernatants after type 14 rhinovirus (RV14), or sham (control) infection. Effects of DEX were examined at maximal production of each cytokine after RV14 infection. Results are reported as means ± S.E.M. from 7 samples. Significant difference from corresponding control values are indicated by ∗*P *< 0.05, **P < 0.01, ***P < 0.001. Significant difference from RV infection alone are indicated by ^++^ *P* < 0.01 (Cited from [36]).

We showed that the corticosteroid dexamethasone, which has been used in rescue therapy for the treatment of asthma exacerbation, inhibits infection by RV14 by reducing the expression of ICAM-1, the receptor for the major RVs, in human tracheal epithelial cells [36]. Dexamethasone also reduced the production of cytokines in epithelial cells [36] (Figures 3 and 5, Table 2).

**Figure 6:**
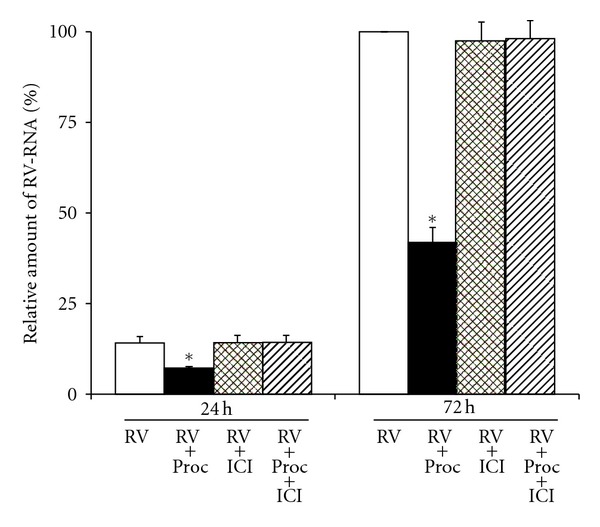
Replication of viral RNA in human tracheal epithelial cells at 1 day (24 h) or 3 days (72 h) after infection with type 14 rhinovirus in the presence of procaterol (0.1 *μ*M) (RV + Proc), vehicle (0.01% ethanol) (Control; RV), ICI 118551 (1 *μ*M) (RV + ICI) or the presence of procaterol (0.1 *μ*M) plus ICI 118551 (1 *μ*M) (RV + Proc + ICI) as detected by real-time quantitative RT-PCR. The epithelial cells isolated from the same donors were treated with either procaterol, vehicle, ICI 118551, or procaterol plus ICI 118551. The results are expressed as the relative amount of RNA expression (%) compared with that of maximal rhinovirus RNA at day 3 (72 h) in the cells treated with vehicle, and reported as means ± S.E.M. from 5 samples (2 ex-smokers and 3 non-smokers). Significant differences from treatment with a vehicle (RV) at each time are indicated by *P < 0.05. (Cited from [39]).

Yamaya et al. demonstrated that the *β*_2_ agonist procaterol reduced RV14 release and RV RNA replication in human tracheal epithelial cells through the reduced expression of ICAM-1 and the increased pH in the endosomes from which the RV RNA enters the cytoplasm [39] (Figures 3 and 6). Procaterol also reduced the RV14 infection-induced release of IL-1*β*, IL-6, and IL-8 [39] (Table 2), whereas salmeterol and the short-acting *β*_2_ agonist salbutamol enhanced the RV-induced IL-6 production in a report by Edwards et al. [34]. Although the precise reason for the different responses to salmeterol, salbutamol, and procaterol is uncertain, the cell types and culture media were different between our study [39] and that by Edwards et al. [34]. As previously reported [118], the differences in the factors in the culture medium might be associated with different responses to drugs. Further studies are needed to define the effects of long-acting *β*_2_ agonists using other cells, such as the primary cultures of human airway epithelial cells, which are cultured in other media. 

We reported the inhibitory effects of several agents on RV14 infection, including a long-acting anticholinergic agent, tiotropium [40], a mucolytic agent, L-carbocisteine [38], a proton pump inhibitor, lansoprazole [45], and a traditional Japanese herbal medicine, hochu-ekki-to [46], by reducing ICAM-1 expression and increasing the pH in endosomes (Figure 3). Additionally, these agents modulate pro-inflammatory cytokines (Table 2). The treatment with L-carbocisteine has been reported to reduce the frequency of common colds and exacerbations in COPD patients [119, 120]. Thus, various drugs that are used to treat obstructive and/or inflammatory lung diseases or other inflammatory diseases may have antiviral effects in addition to their anti-inflammatory effects. 

In a recent report by Koetzler et al. [47], NO was shown to inhibit the RV infection-induced production of CXCL10 (IP-10) by inhibiting the viral activation of NF-*κ*B and the IFN response factors (IRFs) using primary human bronchial epithelial cells or BEAS-2B cells. Cakebread et al. [48] demonstrated the inhibitory effects of IFN-*β* on RV infection and infection-induced CXCL10, RANTES, and IL-6 expression in primary bronchial epithelial cells. Further studies are needed to show the clinical effects of these agents on the modulation of RV infection.

### 8.2. Inhibitory Effects of Macrolides on Rhinovirus Infection

The specific vacuolar H^+^-ATPase inhibitor and the macrolide antibiotic bafilomycin A_1_ [121] blocks the uncoating of the minor RV subgroup, RV2, and the major RV subgroup, RV14, from late endosomes [122, 123]. Suzuki et al. showed the inhibitory effects of bafilomycin A_1_ on RV infection in human tracheal epithelial cells [89]. Bafilomycin A_1_ reduced the viral titer of RV14 and inhibited the production of cytokines, including IL-1*β*, IL-6, IL-8, and TNF-*α*, and ICAM-1 before and after RV14 infection. Bafilomycin A_1_ reduced the susceptibility of epithelial cells to RV14 infection. RV14 increased the levels of activated NF-*κ*B in the cells, and bafilomycin A_1_ reduced the levels of activated NF-*κ*B. Bafilomycin A_1_ decreased the number of acidic endosomes in the epithelial cells.

Furthermore, erythromycin, a clinically used macrolide antibiotic, reduces the supernatant RV14 titer, RV14 RNA levels, susceptibility to RV14 infection, and production of ICAM-1 and pro-inflammatory cytokines [37] (Figure 3, Table 2). Erythromycin also reduces the supernatant RV2 titers, RV2 RNA levels, susceptibility to RV2 infection, and pro-inflammatory cytokine production. Erythromycin reduced the NF-*κ*B activation by RV14 and decreased the number of acidic endosomes in the epithelial cells. These results suggest that the macrolide antibiotics erythromycin and bafilomycin A_1_ inhibit infection by the major RV subgroup by reducing ICAM-1 levels and by both the major and minor RV subgroups by blocking RV RNA entry into the endosomes in human tracheal epithelial cells [37, 88]. These anti-inflammatory effects of the macrolides may be associated with the reduction in the levels of IL-8 and neutrophil elastase in the sputa of refractory asthma patients treated with clarithromycin [124]. Furthermore, macrolides were shown to reduce the frequency of common colds and COPD exacerbation [125–129]. Therefore, the treatment with macrolides is expected to inhibit RV infection and the infection-induced airway inflammation in COPD and refractory asthma, although several issues remain to be resolved, such as bacterial colonization. 

### 8.3. Inhibition of Influenza Virus Infection and Infection-Induced Mediator Release

With regard to the inhibition of the influenza virus, Ochiai et al. [130] demonstrated that bafilomycin A_1_ inhibits the growth of the type A and type B human influenza viruses in Madin Darby Canine Kidney (MDCK) cells. We also reported that clarithromycin and L-carbocisteine reduce viral release and RNA replication of the type A seasonal influenza virus (H_3_N_2_) partly through the reduced expression of the receptor for the human influenza virus in human airway epithelial cells via the inhibition of NF-*κ*B and by increasing the pH of endosomes [55, 56] (Figure 3). Clarithromycin and L-carbocisteine also reduced the influenza virus infection-induced production of IL-1*β*, IL-6 and IL-8 in human tracheal epithelial cells (Table 2). The modulation of influenza virus infection-induced inflammation may be important to improve the condition of the patients. The clinical benefits of these agents in influenza infection are expected, and further studies are required. 

### 8.4. Inhibition of RS Virus Infection and Infection-Induced Mediator Release

Macrolides inhibit RS virus infection partly through the reduced expression of the F protein receptor, activated RhoA, and the inhibition of subsequent Rho kinase activation in human airway epithelial cells (Figure 3). Bafilomycin A_1_ and clarithromycin reduce RS viral titers in the supernatants of cultured cells, the levels of RS viral RNA, the susceptibility of the cells to RS viral infection, and the levels of cytokines induced by RS viral infection [61]. L-carbocisteine also inhibits RS viral infection through the reduced expression of an RS virus receptor, ICAM-1 [77]. These agents may modulate the RS viral infection and the infection-induced airway inflammation. 

## 9. Conclusion

Respiratory viral infections may exacerbate asthma through several mechanisms, including airway inflammation, mucus hypersecretion, and bronchial hyperresponsiveness. Recent reports have demonstrated the association between impaired immune responses and asthma exacerbation during viral infection. The modulation of NF-*κ*B is a promising target for the development of anti-inflammatory therapies that can be used to treat the asthma exacerbation induced by respiratory viral infections. In addition to the development of vaccines and anti-viral drugs for the treatment of RV and RS viruses, the development of anti-inflammatory therapies is required for the treatment and prevention of the asthma exacerbation induced by respiratory viral infections.

## Figures and Tables

**Table:** (a)

Viruses	Samples and specimens	Reference number	Cytokines, mediators, and substances
RV	Nasal secretion and sputum	[13, 25]	IL-6, CXCL8 (IL-8), myeloperoxidase
		[26, 27]	ECP
	Plasma or serum	[18, 19]	Histamine, IL-6, ECP, sICAM-1
	Urine	[19]	LTE_4_
	Exhaled air	[11]	NO

Flu	Nasal secretion	[25]	CXCL8 (IL-8), myeloperoxidase
	Plasma or serum	[19, 28]	Histamine, IL-6, CXCL8 (IL-8), RANTES, ECP, sICAM-1
	Urine	[19]	LTE_4_
	Exhaled air	[29, 30]	CO

URTIs or ARI	Nasal secretion or serum	[31, 32]	IL-6, IL-11

RV: rhinovirus; Flu: influenza virus; RSV: respiratory syncytial virus; IL: interleukin; URTIs: upper respiratory tract infections; ARI: acute respiratory infection. Other nonstandard abbreviations are described in the text.

**Table:** (b)

Viruses	Sample(s) and specimen(s)	Reference number	Cytokines, mediators, and substances
RV	Airway and lung epithelial cells	[12–15, 22, 23, 32–48]	IL-1, IL-6, CCL5 (RANTES), CCL11 (Eotaxin ), CXCL5 (ENA-78), CXCL8 (IL-8), CXCL10 (IP-10), IL-11, TNF-*α*, GM-CSF, IFN-*β*, IFN-*λ*
		[14, 15, 35–37, 49]	ICAM-1, LDL-R, FGF, VEGF, mucin
	Other cells		
	Eosinophils, other leukocyte, and Mϕ	[17, 23, 24, 50–54]	Histamine, IFN-*α*, IFN-*γ*, IFN-*λ*, IL-4, IL-6, IL-10, TNF-*α*
	Mast cell	[53]	Histamine, CXCL8 (IL-8), GM-CSF
	Smooth muscle and fibroblast	[13, 21, 32]	IL-1*β*, IL-5, IL-6, ICAM-1

Flu	Airway and lung epithelial cells	[55, 56]	IL-1, IL-6, CXCL8 (IL-8)
	Other cells	[16, 17, 57, 58]	IL-1*β*, IL-6, TNF-*α*, histamine, protease, IFN-*α*, IFN-*γ*

RSV	Airway and lung epithelial cells and other cells	[17, 32, 59–61]	IL-1, IL-4, IL-6, CCL5 (RANTES), CXCL8 (IL-8), IL-11, GM-CSF, TNF-*α*, histamine

RV: rhinovirus; Flu: influenza virus; RSV: respiratory syncytial virus; IL: interleukin; Mϕ: macrophage. Other nonstandard abbreviations are described in the text.

**Table 2:** Inhibitory effects of drugs or agents on the production of cytokines, mediators, and substances induced by virus infection.

Viruses	Sample(s) and specimen(s)	Reference number	Cytokines, mediators, and substances
RV	Corticosteroids		
	Fluticasone	[33]	CCL5 (RANTES), CXCL8 (IL-8), CXCL10 (IP-10)
		[34]	IL-6
	Budesonide	[35]	CCL5 (RANTES), CXCL8 (IL-8), CXCL10 (IP-10), IL-6, FGF, VEGF
	Dexamethasone	[36]	IL-1, IL-6, L-8, TNF-*α*, ICAM-1
	*β*_2_ agonists		
	Salmeterol	[33]	CCL5 (RANTES), CXCL10 (IP-10)
	Formoterol	[35]	CXCL8 (IL-8), FGF
	Procaterol	[39]	IL-1, IL-6, L-8, ICAM-1
	Anticholinergics		
	Tiotropium	[40]	IL-1, IL-6, L-8, ICAM-1
	Other drugs or agents		
	Erythromycin, carbocisteine, lansoprazole, or hochu-ekki-to	[37, 38] [45, 46]	IL-1, IL-6, L-8, TNF-*α*, ICAM-1
	Nitric oxide	[47]	CXCL10 (IP-10)
	IFN-*β*	[48]	IL-6, CCL5 (RANTES), CXCL10 (IP-10)

Flu	Clarithromycin or carbocisteine	[55, 56]	IL-1, IL-6, L-8

RSV	Clarithromycin or carbocisteine	[61, 77]	IL-1, IL-6, L-8

RV: rhinovirus; Flu: influenza virus; RSV: respiratory syncytial virus; IL: interleukin.

Other nonstandard abbreviations are described in the text.
